# CoRe: Joint Optimization with Contrastive Learning for Medical Image Registration

**DOI:** 10.3390/s26113425

**Published:** 2026-05-28

**Authors:** Eytan Kats, Christoph Grossbroehmer, Ziad Al-Haj Hemidi, Fenja Falta, Wiebke Heyer, Mattias P. Heinrich

**Affiliations:** Insitute of Medical Informatics, University of Luebeck, 23562 Luebeck, Germany; e.kats@student.uni-luebeck.de (E.K.); f.falta@uni-luebeck.de (F.F.);

**Keywords:** image registration, contrastive learning, equivariance

## Abstract

Medical image registration is a fundamental task in medical image analysis, enabling the alignment of images from different modalities or time points. However, intensity inconsistencies and nonlinear tissue deformations pose significant challenges to the robustness of registration methods. Recent approaches leveraging self-supervised representation learning show promise by pre-training feature extractors to generate robust anatomical embeddings, that further used for the registration. In this work, we propose a novel framework that integrates equivariant contrastive learning directly into the registration model. Our approach leverages the power of contrastive learning to learn robust feature representations that are invariant to tissue deformations. By jointly optimizing the contrastive and registration objectives, we ensure that the learned representations are not only informative but also suitable for the registration task. We evaluate our method on abdominal and thoracic image registration tasks, including both intra-patient and inter-patient scenarios. Experimental results demonstrate that the integration of contrastive learning directly into the registration framework significantly improves performance, surpassing strong baseline methods.

## 1. Introduction

Medical image registration is a fundamental problem in medical image analysis, aiming to establish dense anatomical and semantic correspondences between images. These images may be acquired at different time points, from different subjects, or using different imaging modalities. Accurate registration is a prerequisite for a wide range of downstream clinical and research tasks. It enables clinicians and researchers to track disease progression over time, evaluate the effectiveness of therapeutic interventions, quantify structural or functional changes, and analyze anatomical variability across patient populations.

The primary challenges in medical image registration arise from variability in image appearance and complex nonlinear anatomical deformations. Intensity variations caused by differences in imaging protocols, scanner hardware, or acquisition modalities often make direct voxel-wise comparisons unreliable. In addition, anatomical structures may undergo substantial non-rigid deformations due to inter-subject variability, disease progression, respiration, or surgical interventions. These factors complicate the estimation of accurate spatial correspondences and motivate the development of robust registration frameworks capable of handling both appearance changes and large deformations.

Recent advances in self-supervised contrastive learning have shown strong potential for addressing these challenges in medical imaging [1,2,3,4,5]. Contrastive objectives enable networks to learn semantically meaningful voxel-wise representations that remain robust to appearance variability and anatomical deformations. Existing registration approaches typically adopt a two-stage training strategy. First, a feature extractor is pretrained using a contrastive objective independently of the registration task. Second, the pretrained encoder is frozen and used to generate features for registration optimization. While effective, this decoupled design does not explicitly align feature learning with the downstream registration objective.

**Figure 1:**
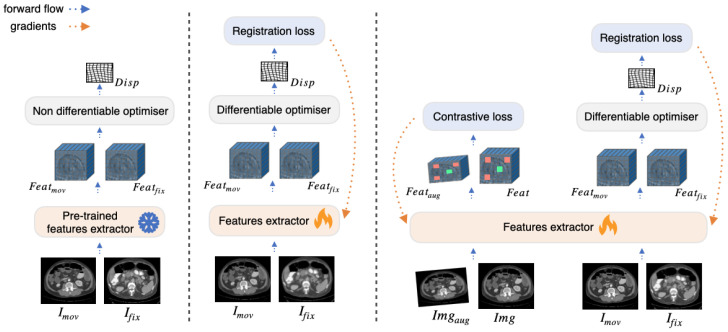
Comparison of hybrid registration methods. From left to right: Feature extractor pretrained separately and used without further optimization during registration (SAMConvex [1]); Feature extractor optimized exclusively with a registration loss during training (Bigalke et al. [6]); Proposed CoRe method, where the feature extractor is jointly optimized under both registration and contrastive loss objectives to enhance feature robustness and registration accuracy.

In this work, we introduce CoRe (**Co**ntrastive learning for medical image **Re**gistration) (The code is available at https://github.com/EytanKats/reg-ssl, accessed on 23 May 2026), a framework that jointly optimizes deformable image registration and self-supervised contrastive learning. Building upon the hybrid registration framework of Bigalke et al. [6], CoRe incorporates a self-supervised equivariant contrastive loss into the training objective. This enables online joint optimization of representation learning and deformable registration within a unified training process. In contrast to prior contrastive registration approaches such as SAMConvex [1], which rely on separately pretrained and frozen feature extractors, CoRe does not require a dedicated pretraining stage and instead continuously adapts the learned feature representations to the downstream registration objective throughout training. The differences between the approaches are illustrated in Figure 1.

The primary contributions of this work are as follows:

- We propose a joint optimization strategy that integrates an online self-supervised equivariant contrastive objective directly into a deformable registration framework.
- We show that jointly optimizing contrastive and registration objectives yields improved registration accuracy compared to separate pretraining or registration-only optimization.
- We evaluate the proposed approach on abdominal and thoracic CT registration benchmarks in both inter-patient and intra-patient settings, demonstrating competitive performance against conventional, learning-based, and hybrid registration methods.

## 2. Related Work

**Structural image representations**: Traditional structural representation methods [7,8,9,10] aim to extract anatomical descriptors that are more robust to intensity variations than raw image intensities. These hand-crafted representations capture local structural patterns while reducing sensitivity to acquisition differences across modalities or scanners. Registration algorithms subsequently estimate spatial transformations by comparing descriptor similarity rather than raw intensities.

**Supervised metric learning**: Deep metric learning approaches replace hand-crafted descriptors with learned feature representations optimized to minimize distances between corresponding anatomical locations in aligned image pairs [11]. Such methods can capture complex anatomical characteristics and tissue variability more effectively than manually designed descriptors. However, they require accurately aligned training data, which is expensive and difficult to obtain in medical imaging applications.

**Self-supervised contrastive learning in medical imaging**: Self-supervised contrastive learning has recently emerged as a powerful paradigm for representation learning in medical imaging [12,13,14,15,16]. By maximizing agreement between augmented views of the same image, contrastive learning enables models to learn semantically meaningful representations without requiring manual annotations. Data augmentation plays a critical role in this process. Intensity augmentations encourage invariance to appearance variations, whereas geometric transformations such as rotations, scaling, and elastic deformations promote robustness to spatial variability. More recent work has incorporated equivariance constraints into contrastive objectives [2,17,18], ensuring that transformations in the input space induce predictable transformations in the embedding space. Such equivariant representations are particularly relevant for deformable registration, where anatomical structures undergo spatial transformations.

**Contrastive learning for medical image registration**: Recent studies have demonstrated that contrastive learning can generate dense feature representations well suited for deformable registration [1,2,3,4,5]. Existing approaches mainly differ in how feature extraction is integrated with deformation estimation.

Some methods extract features after deformation estimation. Mok et al. [4] pretrain a feature extractor using contrastive learning and subsequently apply a mean squared error loss between features extracted from fixed and warped moving images during registration. Similarly, ContraReg [5] applies a contrastive objective to dense multi-scale feature maps extracted from fixed and warped images using a pretrained autoencoder. In both approaches, the feature extractor is pretrained independently and remains frozen during registration training.

Other methods extract features prior to deformation estimation and use them as inputs to the registration framework. CoMIR [2] employs supervised contrastive learning on aligned multimodal image pairs to map images into a shared latent space, followed by separate registration training. SAMConvex [1] and SAME [3] leverage Self-supervised Anatomical eMbeddings (SAM) [15] for registration. SAMConvex combines SAM embeddings with convex optimization strategies [19], while SAME integrates SAM features into a VoxelMorph-based registration framework [20].

CoRe follows the pre-deformation feature extraction paradigm, where features are extracted independently from the fixed and moving images prior to deformation estimation and subsequently processed by a differentiable optimization module to infer the deformation field. In contrast to previous approaches that rely on independently pretrained embeddings, CoRe jointly optimizes feature learning and deformable registration within a unified framework by integrating a self-supervised equivariant contrastive objective directly into the registration process. This joint optimization enables the learned representations to remain robust to tissue deformations while being specifically tailored for accurate deformation estimation.

## 3. Materials and Methods

### 3.1. Problem Definition

Let $I_{f},I_{m}$ denote the fixed and moving images, respectively. The training dataset consists of |Ω| image pairs $\Omega ={\left\{I_{f}^{s},I_{m}^{s}\right\}}_{s=1}^{\left|\Omega \right|}$. The registration framework R comprises a trainable feature extractor G and a deterministic optimization module H. Given $I_{f}$ and $I_{m}$ it predicts a displacement field $u=R(I_{f},I_{m})$. Ideally, the intensity values $I_{f}\left(p\right)$ and $\left[Su\circ I_{m}\right]\left(p\right)$ should correspond to the same anatomical location, where $\left[Su\circ I_{m}\right]$ represents $I_{m}$ warped by the spatial transformation Su induced by *u*. The objective is to train G to extract high-quality features, enabling H to compute an optimal displacement field for accurate image alignment.

**Figure 2:**
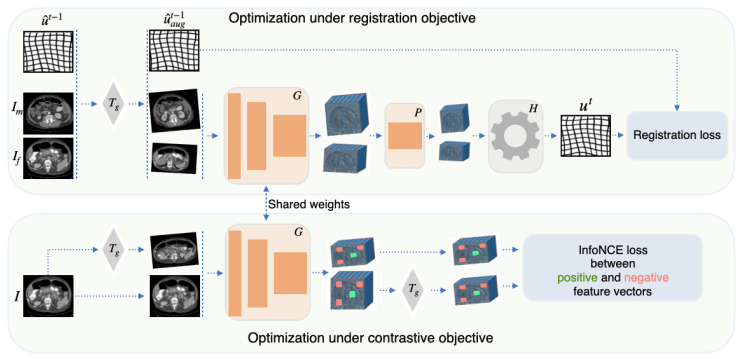
Overview of the proposed CoRe framework: The feature extractor is jointly optimized using registration and equivariance-based contrastive objectives, enabling robust and spatially coherent feature representations for precise displacement field estimation. The dotted arrows indicate the flow of data through the registration framework during the training process.

In this work, we incorporate equivariance constraints, formulated through a contrastive objective (Section 3.3), directly into the registration framework (Section 3.2). This integration ensures that the internal feature representations corresponding to identical anatomical locations remain robust to tissue deformations. Figure 2 presents an overview of the proposed joint optimization strategy, highlighting the simultaneous optimization of the feature extractor under both contrastive and registration objectives. Algorithm 1 outlines the pseudo-code for the training procedure, detailing the steps involved in leveraging the synergistic interaction between these two objectives to enhance registration accuracy and robustness.
**Algorithm 1:** Joint training procedure of CoRe for a single stage *t*.
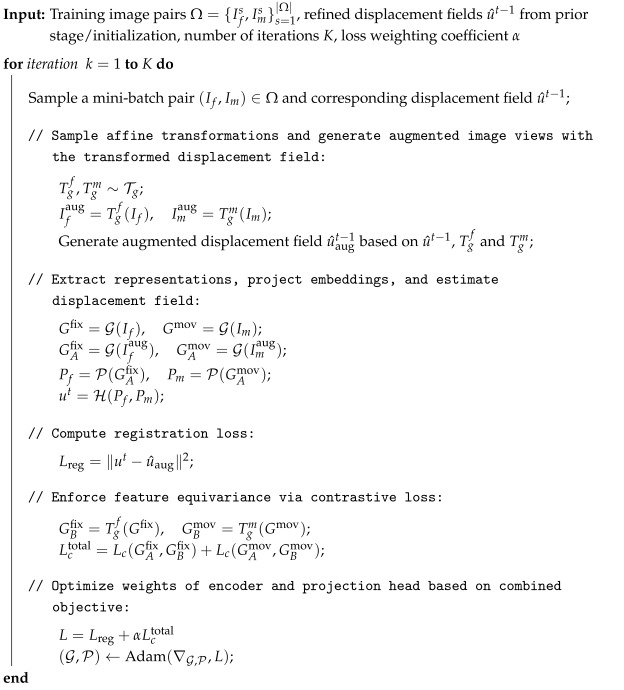


### 3.2. Registration Framework

We use a hybrid registration pipeline comprising a convolutional feature extractor G, a convolutional projection head P, and a differentiable optimization module H, that infers a displacement field from the fixed and moving features. The optimization module H employs a differentiable version of the coupled convex-discrete optimization framework [19]. The optimization begins by constructing a 6D correlation volume over a discrete mesh grid of relative displacements, computing the feature similarities between the projected fixed embeddings $P_{f}$ and moving embeddings $P_{m}$. Next, a quadratic penalty term is added along the displacement dimensions of the cost volume to act as a regularizing coupling term. Finally, to enable end-to-end backpropagation, the traditional non-differentiable argmin operator is replaced with a softmin function across the displacement dimensions, followed by a point-wise multiplication with the discrete grid to compute a continuous expectation of displacements.

The pipeline begins with G extracting feature representations, $G(I_{f})$ and $G(I_{m})$, from the fixed and moving images, respectively. These representations are then passed through the projection head P, and the resulting embeddings are processed by the optimizer H, which predicts the displacement field *u* as follows:

(1) $$u=R\left(I_{f},I_{m}\right)=H\left(P\left(G\left(I_{f}\right)\right),P\left(G\left(I_{m}\right)\right)\right).$$

We adopt the self-training scheme with pseudo-labels [6] as a strong baseline for deformable image registration. Training proceeds in *M* stages. At the beginning of each stage t=1,…,M, the registration pipeline R generates displacement fields $u^{t-1}$ for all image pairs. These fields are refined through an instance optimization process comprising three key steps [6]. First, a forward-backward consistency check is applied by estimating both the forward displacement field $u_{m\to f}$ and the backward field $u_{f\to m}$, and subsequently minimizing the discrepancy between them. Second, a double warping procedure is employed, which warps the moving image with the inferred displacement field prior to repeating the registration steps. Third, an instance optimization loop is executed for a fixed number of iterations per image pair to jointly minimize the regularization cost and feature dissimilarity.

Pseudo-label generation is performed on the original image pairs without data augmentation. During stage *t* training, affine augmentations are applied to the input images, and the corresponding pseudo-labels are transformed accordingly to obtain augmented displacement fields ${\hat{u}}_{\mathrm{aug}}^{t-1}$, ensuring consistency between the supervision signal and the augmented image pairs. At the start of training, pseudo-labels ${\hat{u}}^{0}$ are generated using randomly initialized G and P.

The training objective minimizes the mean squared error (MSE) loss between the displacement fields $u^{t}$ predicted at training step of stage *t* and the pseudo-labels ${\hat{u}}^{t-1}$ generated at the beginning of that stage:

(2) $$L_{\mathrm{reg}}={∥u^{t}-{\hat{u}}^{t-1}∥}^{2}.$$

To enhance the diversity of transformations during training, augmentation is applied to the pseudo displacement field (Figure 2). Specifically, the fixed and moving images, $I_{f}$ and $I_{m}$, are each transformed using unique random affine augmentations $T_{g}^{f}$ and $T_{g}^{m}$, respectively. The pseudo displacement field $\hat{u}$ is then adjusted to account for these affine transformations, resulting in the augmented displacement field ${\hat{u}}_{aug}$.

### 3.3. Equivariance Constraint

The quality of the displacement field *u* generated by the optimizer H relies on the quality of the features extracted by the network G. Ideally, the embeddings produced by G for the same anatomical location in the moving image $I_{m}$ and the fixed image $I_{f}$ should be identical, regardless of geometric deformations between the images. Such consistency in feature embeddings provides H with a robust initialization, allowing it to generate accurate displacement fields.

While the registration loss $L_{\mathrm{reg}}$ (Section 3.2) naturally improves the features extracted by G during training, we propose incorporating a contrastive objective to further refine feature quality. Specifically, to address the challenges posed by geometric deformations in tissue, we introduce an equivariance constraint on the image embeddings.

This constraint enforces consistency between embeddings derived from the same image *I* under geometric transformations. We apply an affine transformation $T_{g}∼T_{g}$, sampled from a predefined augmentation set $T_{g}$, to the image *I*. The constraint enforces consistency between the transformed features of the original image, $G_{A}=T_{g}\left(G\left(I\right)\right)$, and the features extracted from the transformed image, $G_{B}=G\left(T_{g}\left(I\right)\right)$. Note that the objective enforces geometric equivariance rather than invariance. Consequently, the model is encouraged to satisfy $T_{g}\left(G\left(I\right)\right)\approx G\left(T_{g}\left(I\right)\right)$, ensuring that geometric transformations in the input space induce corresponding transformations in the feature space. By enforcing this property, the model learns representations that are robust to tissue deformations - an essential requirement for registration tasks, where features must remain consistent across anatomical distortions.

We implement the equivariance constraint using an InfoNCE loss [21] applied to feature vectors extracted from corresponding spatial locations in the feature maps $G_{A}$ and $G_{B}$. Let $f_{A}^{j}$ and $f_{B}^{j}$ denote feature vectors sampled from the *j*th spatial location in $G_{A}$ and $G_{B}$, where j=1,…,n. Each feature vector $f_{A}^{j}$ forms one positive pair with the corresponding vector $f_{B}^{j}$ and 2·(n−1) negative pairs with other feature vectors sampled from $G_{A}$ and $G_{B}$. The contrastive loss is then defined as:

(3) $$L_{c}=-\sum_{j}\log\frac{d(f_{A}^{j},f_{B}^{j})}{d\left(f_{A}^{j},f_{B}^{j}\right)+\sum_{l\neq j}\sum_{k\in A,B}d\left(f_{A}^{j},f_{k}^{l}\right)},$$

where $d\left(f_{A}^{j},f_{B}^{j}\right)=\exp\left(\langle f_{A}^{j},f_{B}^{j}\rangle /\tau \right)$, 〈·,·〉 denotes the inner product, and τ is a temperature scaling factor that controls the sharpness of the similarity distribution. In our experiments, we set τ=0.1.

During training, the equivariance constraint is applied independently to the fixed and moving images. The total contrastive loss is therefore computed as the sum of the losses evaluated on the corresponding feature pairs:

(4) $$L_{c}^{\mathrm{total}}=L_{c}\left(G_{A}^{\mathrm{fix}},G_{B}^{\mathrm{fix}}\right)+L_{c}\left(G_{A}^{\mathrm{mov}},G_{B}^{\mathrm{mov}}\right),$$

where $\left(G_{A}^{\mathrm{fix}},G_{B}^{\mathrm{fix}}\right)$ and $\left(G_{A}^{\mathrm{mov}},G_{B}^{\mathrm{mov}}\right)$ denote the feature pairs constructed from the fixed and moving images, respectively.

### 3.4. Joint Optimization

By jointly minimizing the registration loss (Section 3.2) and the contrastive loss (Section 3.3), the proposed framework ensures that the feature representations extracted by the network G are robust to geometric transformations while remaining well-suited to the optimization procedure defined by the optimizer H. The registration loss drives the alignment of fixed and moving images, encouraging the feature extractor to generate embeddings that are specifically tailored for consumption by the optimizer. Concurrently, the contrastive loss provides valuable guidance to the optimization process by imposing equivariance to geometric distortions, fostering the consistency of feature embeddings for same anatomical locations in registered images. The joint optimization process integrates the strengths of both losses, improving the robustness and accuracy of the registration framework. The combined loss function is defined as:

(5) $$L=L_{reg}+\alpha \cdot L_{c}^{\mathrm{total}},$$

where α is a weighting coefficient that balances the contributions of the contrastive loss and the registration objective.

### 3.5. Implementation Details

The feature extractor G consists of four convolutional blocks with 3×3×3 convolutions, batch normalization, and ReLU activations. The projection head P consists of a single convolutional block with 128 output channels, a kernel size of 3×3×3, and a stride of 2, followed by a final convolutional layer with a kernel size of 1×1×1, which projects the feature maps to 16 channels. The framework is trained for M=8 stages, with each stage consisting of 1000 iterations and a batch size of 2. Optimization is performed using the Adam optimizer, and the learning rate follows a cosine annealing warm restart schedule, decaying from $1\times 10^{-3}$ to $1\times 10^{-5}$. The contrastive loss is applied to the output of the final block’s convolutional layer, with 1000 feature vectors sampled per image pair. All training and inference experiments were conducted on a single NVIDIA A100 GPU (NVIDIA Corporation, Santa Clara, CA, USA).

### 3.6. Datasets

We evaluate the performance of the proposed method on the challenging inter-patient abdominal CT registration dataset [22]. This dataset comprises 30 3D abdominal CT scans from different patients, with 13 manually labeled anatomical structures: spleen, right kidney, left kidney, gall bladder, esophagus, liver, stomach, aorta, inferior vena cava, portal and splenic vein, pancreas, left adrenal gland, and right adrenal gland. All images are resampled to a uniform voxel resolution of 2 mm and standardized to spatial dimensions of 192×160×256 voxels. The training-test split of this dataset defined in Learn2Reg challenge [23] widely adapted in the medical image registration community which facilitates direct comparison with prior works. Specifically, the training set includes 20 scans (190 image pairs), while the test set consists of 10 scans (45 image pairs).

To evaluate performance in the intra-patient setting, we utilize the RAD-ChestCT dataset [24]. In this dataset, we identified 371 longitudinal scan pairs. We split the data to 300 pairs designated for training and 71 pairs for testing. The CT images are resampled to a consistent voxel resolution of 1.5 mm and spatial dimensions of 256×256×224 voxels. Since the RAD-ChestCT dataset does not include manual segmentation labels, we employ the TotalSegmentator tool [25] to segment the CT scans. Using the resulting segmentations, we calculate registration accuracy across 22 anatomical structures: 5 lung lobes, vertebrae from T1 to T12, heart myocardium, left and right heart ventricles and atriums.

## 4. Results and Discussion

To assess accuracy of the registration, we compute the average Dice similarity coefficient (DSC) using available segmented structures. The plausibility of the deformation fields is evaluated using the standard deviation of the logarithm of the Jacobian determinant (SDlogJ). Additionally, we report inference run-time ($T_{inf}$) across methods.

### 4.1. Registration Results

We compare our method with conventional registration approaches (NiftyReg [26] and DEEDs [27]), learning-based methods (VoxelMorph [20], LapIRN [28] and uniGradIcon [29]), and two hybrid approaches (Bigalke et al. [6] and SAMConvex [1]) (Table 1). NiftyReg uses multi-resolution optimization with mutual information, while DEEDs relies on edge-based similarity with B-spline deformation. VoxelMorph and LapIRN directly regress dense displacement fields using convolutional neural networks, with LapIRN incorporating multi-scale refinement. uniGradICON improves robustness via gradient inverse consistency (GradICON [30]) and is trained on a diverse collection of data. During inference, we employ the instance-specific optimization option provided by uniGradICON, which fine-tunes the pretrained model weights for each image pair to achieve improved performance. Bigalke et al. and SAMConvex are hybrid approaches that leverage CNNs for feature extraction from image pairs and classical optimization techniques for displacement field estimation. SAMConvex uses a pretrained SAM model [15] for feature extraction, while Bigalke et al. optimize the feature extractor with a differentiable optimizer and registration loss (Figure 1).

**Figure 3:**
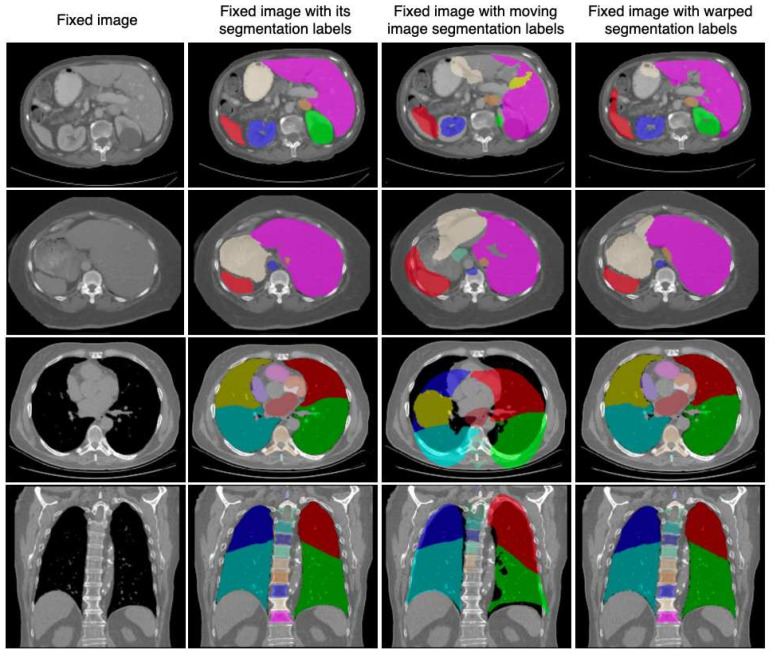
Qualitative results of the proposed CoRe method. From **left** to **right**: fixed image, fixed image with its segmentation overlay, fixed image with the overlay of the moving image segmentation, and fixed image with the overlay of the warped segmentation. The top two rows show examples from the AbdomenCT dataset in the axial plane, while the bottom two rows present examples from the RadChestCT dataset in axial and coronal planes. Different colors correspond to different anatomical structures, illustrating the deformations of each structure.

VoxelMorph and LapIRN are computationally efficient, however, they often underperform compared to traditional and hybrid methods. uniGradICON achieves strong results on both datasets, but relies on instance-specific optimization during inference, which leads to longer inference times. DEEDS achieves strong results on the RadChestCT dataset, ranking as the second-best method. This performance is expected due to its focus on optimizing edge similarity, which is highly effective for intra-patient thoracic datasets where edges in image pairs align closely. However, on the AbdomenCT dataset, where deformations between image pairs are more complex, DEEDS demonstrates lower accuracy compared to hybrid methods. Hybrid approaches combine deep learning’s ability to extract robust features with the precision and reliability of classical optimization techniques for displacement field estimation. This synergy enables hybrid methods to achieve state-of-the-art performance on the challenging inter-patient AbdomenCT dataset while maintaining competitive results on RadChestCT. Our proposed CoRe method achieves the best performance on both datasets, delivering the highest Dice scores (DSC) while preserving smoothness in the predicted displacement fields (SDLogJ), comparable to competitive methods. These results underscore the effectiveness of our approach, which incorporates an equivariance-based contrastive objective directly into the registration framework, enabling performance improvement for image registration tasks. Figure 3 presents qualitative registration results of the proposed method on the AbdomenCT and RadChestCT datasets.

### 4.2. Ablations Study

To assess the effectiveness of the proposed method, we trained the feature extractor G using regularization loss and contrastive loss independently and compared the results with the proposed joint optimization approach (Table 2). For the contrastive loss, we initially pretrained G using only contrastive objective and subsequently trained the registration framework with G frozen using only registration objective. The joint optimization approach demonstrates superior performance, underscoring the synergistic benefits of combining these objectives. This strategy facilitates the extraction of more discriminative and spatially coherent features, enhancing registration accuracy across datasets.

Along with the equivariance constraint described in Section 3.3, self-supervised contrastive learning methods commonly employ non-linear intensity augmentations during pretraining to promote feature invariance to appearance changes while preserving spatial encoding. To evaluate their impact, we train our framework with geometric equivariance and appearance invariance constraints independently and jointly (Table 3). Interestingly, the results reveal that within the proposed framework, non-linear intensity augmentations do not provide additional benefits over training solely with the geometric equivariance constraint. For the AbdomenCT dataset, training with intensity augmentations for contrastive loss even results in inferior performance compared to using the registration objective alone. We hypothesize that this is due to the mono-modal nature of the CT datasets used in our evaluation. The standardized intensity values in the CT datasets may limit the effectiveness of intensity augmentations, as they do not enhance the discriminative capacity of the learned features. Future work may explore the utility of intensity augmentations in multi-modal settings or datasets with greater intensity variability, where these augmentations could play a more significant role in improving registration performance.

**Figure 4:**
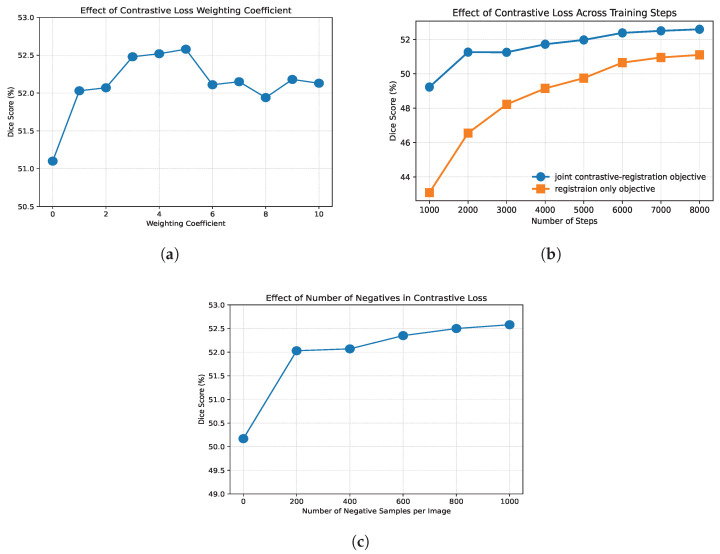
(**a**) Influence of the contrastive loss weighting coefficient α on registration performance. (**b**) Evolution of the Dice score over training iterations, comparing the proposed joint optimization strategy with a baseline trained using only the registration loss. (**c**) Effect of the number of negative samples on registration accuracy. The case of zero negative samples corresponds to training with a cosine similarity loss applied only to positive feature pairs.

We further evaluate the performance of the proposed joint contrastive-registration framework with respect to different values of the weighting coefficient α (Figure 4a), which controls the contribution of the contrastive loss in the total objective (Equation (5)). Incorporating the contrastive component with α=1 already yields a measurable improvement, increasing the Dice score by 0.93% compared to the baseline trained without contrastive supervision. As α increases, we observe a gradual improvement in performance, suggesting that a stronger emphasis on the contrastive objective encourages the learning of more robust and deformation-consistent feature representations. The best performance, with a Dice score of 52.59%, is achieved at α=5, indicating a favorable balance between registration accuracy and representation learning. Increasing α beyond this value leads to a decline in performance, which may indicate that excessive weighting of the contrastive objective can interfere with the optimization of the registration task. Nevertheless, even at higher values of α, the proposed framework consistently outperforms the baseline, supporting the effectiveness of the joint optimization strategy.

Figure 4b illustrates the effect of the contrastive loss across different stages of training. The most pronounced improvement over the baseline is observed during the early training phases, highlighting the impact of contrastive supervision in guiding the optimization process. The contrastive objective provides an informative learning signal at the beginning of training, enabling the model to converge more rapidly toward meaningful feature representations that are beneficial for registration. This is reflected in a performance gap of 6.12% Dice after the first 1000 iterations. After only 2000 iterations, corresponding to one quarter of the total training, the proposed joint optimization strategy already achieves a Dice score of 51.26%, surpassing the final performance of the baseline, 51.1% Dice. Although the performance gap decreases as training progresses, it remains significant throughout the optimization and persists until convergence. These results suggest that integrating contrastive learning not only improves final performance but also contributes to faster convergence.

Negative samples in the contrastive objective act as a regularization mechanism that prevents trivial solutions and promotes the formation of a well-structured latent feature space [21]. In the proposed joint optimization framework, the additional registration objective already constrains the optimization process, reducing the likelihood of convergence to a trivial solution even when no negative samples are used. Nevertheless, training with only a cosine similarity objective, without negative samples, results in inferior performance compared to optimization using only the registration loss (50.2% versus 51.1% Dice on AbdomenCT), indicating insufficient feature discrimination and potential feature collapse. This setting corresponds to the case of zero negative samples in Figure 4c. Furthermore, the number of negative samples has a positive impact on registration accuracy (Figure 4c). Increasing the number of negative samples improves the discriminative capacity of the learned representations, leading to progressively better registration performance. However, these improvements gradually saturate as the number of negative samples increases, while the associated memory consumption grows substantially. This observation suggests that a moderate number of negative samples provides the reasonable trade-off between registration accuracy and computational efficiency.

## 5. Conclusions

We introduced CoRe, a hybrid image registration framework that integrates contrastive learning into the registration pipeline. We demonstrated that jointly optimizing the feature extractor under both contrastive and registration objectives facilitates the learning of semantically coherent and discriminative features, tailored to the requirements of classical optimization procedures. Our findings emphasize the important role of equivariant geometric constraints, implemented through contrastive loss, in enabling the extraction of robust features. These features are particularly effective in handling tissue deformations, thereby improving registration performance. In addition, our analysis shows that the inclusion of contrastive supervision accelerates convergence, especially during the early stages of training, where the model benefits from a stronger and more informative learning signal.

A key limitation of the current study is its exclusive evaluation on mono-modal thoracic and abdominal CT datasets. As indicated by our experimental findings, standard intensity augmentations did not yield significant performance gains, likely due to the standardized Hounsfield Units (HU) in CT imaging, which inherently simplifies intensity mapping. Future work will focus on adapting CoRe to different imaging modalities as well as multi-modal scenarios. This will necessitate investigating contrastive loss strategies capable of accommodating non-linear intensity relationships present when aligning features across different imaging modalities. Furthermore, while offering superior alignment accuracy, CoRe exhibits a higher inference time compared to pure learning-based networks due to the instance optimization step executed after feature extraction. A promising direction for future research involves designing a joint optimization framework that embeds contrastive loss into fully learning-based architectures. This would allow the model to preserve rapid, single-pass inference speeds while potentially boosting overall registration accuracy.

In summary, CoRe indicates that combining contrastive learning with registration objectives offers a promising direction for medical image alignment. By integrating transformation-equivariant feature representations into a registration pipeline, the proposed framework demonstrates an improvement in alignment accuracy for mono-modal CT data. These findings highlight the utility of joint optimization strategies in contributing to more robust and consistent registration workflows.

## Figures and Tables

**Table 1:** Quantitative comparison of registration performance on the AbdomenCT and RadChestCT datasets. DSC denotes Dice similarity coefficient, SDLogJ the standard deviation of the logarithm of the Jacobian determinant used to assess deformation smoothness, and $T_{inf}$ the inference time. Upward arrows (↑) indicate higher-is-better metrics, while downward arrows (↓) indicate lower-is-better metrics. Bold values indicate the best-performing method for each metric and dataset. Statistical significance was evaluated using a paired Wilcoxon signed-rank test between CoRe and the second-best performing method for each dataset (n=45 test image pairs for AbdomenCT and n=71 for RadChestCT). Statistically significant improvements are indicated by p<0.0001.

Method	AbdomenCT			RadChestCT		
	DSC↑	SDLogJ↓	$T_{\inf}↓$	DSC↑	SDLogJ↓	$T_{\inf}↓$
Initial	25.9 ± 7.1	–	–	34.1 ± 14.5	–	–
DEEDs [27]	46.5 ± 8.1	0.058	45.2 s	88.5 ± 6.9	0.049	45.0 s
NiftyReg [26]	34.9 ± 9.3	**0.034**	123.5 s	84.4 ± 7.4	**0.021**	85.5 s
VoxelMorph [20]	35.4 ± 8.9	0.134	**0.2 s**	55.6 ± 10.3	0.101	**0.3 s**
LapIRN [28]	42.4 ± 8.2	0.089	0.7 s	83.4 ± 7.6	0.075	0.8 s
uniGradICON [29]	52.1 ± 6.9	0.117	39.7 s	86.8 ± 8.3	0.092	45.6 s
SAMConvex [1]	51.2 ± 7.8	0.096	5.1 s	88.1 ± 7.1	0.079	6.4 s
Bigalke et al. [6]	51.1 ± 7.3	0.146	1.2 s	87.3 ± 6.9	0.106	1.9 s
CoRe (ours)	**52.6 ± 7.5**	0.148	1.2 s	**89.4 ± 6.6**	0.109	1.9 s

**Table 2:** Comparison of training the feature extractor with registration and contrastive objectives separately and jointly. Upward arrows (↑) indicate higher-is-better metrics, while downward arrows (↓) indicate lower-is-better metrics. Bold values indicate the best-performing method for each metric and dataset. The proposed joint optimization strategy achieves the best performance.

$L_{\mathrm{reg}}$	$L_{\mathrm{cl}}$	AbdomenCT		RadChestCT	
		** DSC↑ **	** SDLogJ↓ **	** DSC↑ **	** SDLogJ↓ **
✓		51.1 ± 7.3	**0.146**	87.3 ± 6.9	**0.106**
	✓	50.3 ± 7.5	0.151	86.7 ± 7.1	0.117
✓	✓	**52.6 ± 7.5**	0.148	**89.4 ± 6.6**	0.109

**Table 3:** Comparison of the effect of intensity invariance and geometric equivariance constraints, applied separately and jointly. Upward arrows (↑) indicate higher-is-better metrics, while downward arrows (↓) indicate lower-is-better metrics. Bold values indicate the best-performing method for each metric and dataset. Training with the equivariance constraint achieves the best performance, highlighting the importance of feature robustness to non-linear tissue deformations.

$T_{i}$	$T_{g}$	AbdomenCT		RadChestCT	
		** DSC↑ **	** SDLogJ↓ **	** DSC↑ **	** SDLogJ↓ **
		51.1 ± 7.3	0.146	87.3 ± 6.9	0.106
✓		49.4 ± 8.1	0.139	87.5 ± 7.0	0.115
	✓	**52.6 ± 7.5**	0.148	**89.4 ± 6.6**	0.109
✓	✓	50.2 ± 7.6	**0.138**	89.3 ± 6.5	**0.102**

## Data Availability

The datasets analyzed in this study are publicly accessible through third-party repositories. The inter-patient abdominal CT registration dataset [26] is available via Zenodo at https://doi.org/10.5281/zenodo.3715652. The RAD-ChestCT dataset is available via Zenodo at https://doi.org/10.5281/zenodo.6406114.
